# CR6 interacting factor 1 deficiency promotes endothelial inflammation by SIRT1 downregulation

**DOI:** 10.1371/journal.pone.0192693

**Published:** 2018-02-23

**Authors:** Shuyu Piao, Jun Wan Lee, Harsha Nagar, Saet-byel Jung, Sujeong Choi, Seonhee Kim, Ikjun Lee, Sung-min Kim, Nara Shin, Yu Ran Lee, Sang Do Lee, Jin Bong Park, Kaikobad Irani, Minho Won, Gang Min Hur, Byeong Hwa Jeon, Dong Woon Kim, Cuk-Seong Kim

**Affiliations:** 1 Department of physiology & Medical Science, School of Medicine, Chungnam National University, Daejeon, Republic of Korea; 2 Emergency ICU, Regional Emergency Center, Chungnam National University Hospital, Daejeon, Republic of Korea; 3 Department of Endocrinology, Chungnam National University Hospital, Daejeon, Republic of Korea; 4 Department of Anatomy & Medical Science, School of Medicine, Chungnam National University, Daejeon, Republic of Korea; 5 Division of Cardiovascular Medicine, Department of Internal Medicine, University of Iowa Carver College of Medicine, Iowa City, IA United States of America; 6 Department of Pharmacology, School of Medicine, Chungnam National University, Daejeon, Republic of Korea; Medical College of Wisconsin, UNITED STATES

## Abstract

**Aims:**

CR6 interacting factor 1 (CRIF1) deficiency impairs mitochondrial oxidative phosphorylation complexes, contributing to increased mitochondrial and cellular reactive oxygen species (ROS) production. CRIF1 downregulation has also been revealed to decrease sirtuin 1 (SIRT1) expression and impair vascular function. Inhibition of SIRT1 disturbs oxidative energy metabolism and stimulates nuclear factor kappa-light-chain-enhancer of activated B cells (NF-κB)-induced inflammation. Therefore, we hypothesized that both CRIF1 deficiency-induced mitochondrial ROS production and SIRT1 reduction play stimulatory roles in vascular inflammation.

**Methods and results:**

Plasma levels and mRNA expression of proinflammatory cytokines (tumor necrosis factor (TNF)-α, interleukin (IL)-1β, and IL-6) were markedly elevated in endothelium-specific CRIF1-knockout mice and CRIF1-silenced endothelial cells, respectively. Moreover, CRIF1 deficiency-induced vascular adhesion molecule-1 (VCAM-1) expression was consistently attenuated by the antioxidant N-acetyl-cysteine and NF-κB inhibitor (BAY11). We next showed that siRNA-mediated CRIF1 downregulation markedly activated NF-κB. SIRT1 overexpression not only rescued CRIF1 deficiency-induced NF-κB activation but also decreased inflammatory cytokines (TNF-α, IL-1β, and IL-6) and VCAM-1 expression levels in endothelial cells.

**Conclusions:**

These results strongly suggest that CRIF1 deficiency promotes endothelial cell inflammation by increasing VCAM-1 expression, elevating inflammatory cytokines levels, and activating the transcription factor NF-κB, all of which were inhibited by SIRT1 overexpression.

## Introduction

Endothelial cells, which are located in the inner monolayer of the blood vessels, play important roles in maintaining vascular homeostasis and regulating the circulatory system [1]. A considerable amount of research has shown that endothelial dysfunction is an early determinant of the development of many cardiovascular diseases such as hypertension, hypercholesterolemia, and atherosclerosis [1–3]. Mitochondria are present in relatively low concentration (2–6%) with low energy requirement in endothelial cells compared to other cell types [4]. Although mitochondria are not the major source of energy production in endothelial cells, they are the main cellular sites of reactive oxygen species (ROS) production and serve as important ROS regulator of intracellular signaling pathways [5, 6]. Many studies have strongly approved that increased mitochondrial ROS production would contribute to endothelial dysfunction in the setting of cardiovascular disease [7, 8]. Endothelial dysfunction in turn promotes vascular inflammation by inducing the production of adhesion molecules, like vascular cell adhesion molecule-1 (VCAM-1) and intercellular cell adhesion molecule-1 (ICAM-1), inflammatory mediators like cytokines, and vasoconstrictors [9].

CR6 interacting factor 1 (CRIF1), a mitochondrial protein, is associated with large mitoribosomal subunits and is essential for normal functioning of the mitochondria [10]. CRIF1 deficiency is a pivotal factor leading to mitochondrial oxidative phosphorylation dysfunction by excessive ROS production [11]. We previously showed that CRIF1 deficiency leads to a considerable increase in mitochondrial ROS production in vascular endothelial cells [12]. Although the role of CRIF1 deficiency is well known for the development of mitochondrial dysfunction, the effect of CRIF1 deletion on vascular inflammation remains unknown.

Silent information regulator factor 2-related enzyme 1 (SIRT1) contributes to cellular regulation and plays an important role in vascular endothelial function by deacetylating proteins [13]. SIRT1 is also well known to protect against endothelial inflammation [14]. Extensive literature indicates that SIRT1 suppresses nuclear factor kappa-light-chain-enhancer of activated B cells (NF-κB) activation by deacetylating the RelA/p65 subunit of the NF-κB complex [15, 16]. On the contrary, inhibition of SIRT1 enhanced inflammation by activating the major inflammatory transcription factor NF-κB [17, 18]. The NF-κB pathway plays a pivotal role in inflammatory phenotype changes under pathophysiological conditions, which can be triggered by cytokines and growth factors, including tumor necrosis factor (TNF)-α, interleukin (IL)-1β, and IL-6, insulin growth factors, and vascular endothelial growth factor [7,8]. We previously revealed that CRIF1 downregulation decreased SIRT1 expression along with increased endothelial nitric oxide synthase acetylation, reducing nitric oxide production [19]. Therefore, we hypothesized that CRIF1 deletion-induced oxidative stress and SIRT1 reduction may stimulate inflammatory responses by inducing NF-κB activation. In this study, we aimed to investigate whether downregulation of CRIF1 triggers inflammation and further to reveal the underlying mechanism.

We proved that CRIF1 deficiency promoted endothelial cell inflammation by increasing the levels of inflammatory cytokines (TNF-α, IL-1 β, IL-6) and VCAM-1 expression via the NF-κB pathway. Also overexpression of SIRT1 led to recovery of the CRIF1 deficiency-induced inflammatory responses in endothelial cells.

## Materials and methods

### Cell culture and transfection

Human umbilical vein endothelial cells (HUVECs) were purchased from Clonetics (San Diego, CA, USA) and cultured in endothelial growth medium 2 from Lonza (Walkersville, MD, USA) according to the manufacturer’s instructions at 37°C with 5% CO_2_. Subconfluent proliferating HUVECs were used at passages 2–8. HUVECs were transfected with small interfering RNA (siRNA) targeting CRIF1 (human siRNA sequence: sense-5'-UGGAGGCCGAAGAACGCGAAUGGUA-3' and antisense-5'-UACCAUUCGCGUUCUU CGGCCUCCA-3'), SIRT 1 (human siRNA sequence: 5'-AAGTACAATCCACTCCGGAA TGA-3' and antisense-5'-GGGCCCCAGGGATGAAG-3'), and negative control siRNA using Lipofectamine 2000 from Invitrogen (Carlsbad, CA, USA) per the manufacturer’s recommendations. The cells were incubated at 37°C in a 5% CO_2_ incubator for 48 h for gene knockdown. Silencing efficiency was measured by western blotting and quantitative polymerase chain reaction (qPCR).

### Mouse studies

All animal studies were conducted at Chungnam National University following the guidelines of the Institutional Animal Use and Care Committee. All experimental procedures were approved by Chungnam National University. Floxed CRIF1 (CRIF1^flox/flox^) mice were generated as described previously [10]. Tek-Cre mice were purchased from Jackson Laboratory (Bar Harbor, ME, USA). To identify the genotype, PCR was performed using specific primers and extracted genomic DNA from tail snips. Mice were maintained in a controlled environment (12-h light/dark cycle; humidity 50–60%; ambient temperature 23°C) and fed chow (Harlan; Indianapolis, IN, USA) *ad libitum*. All animal studies were performed in the animal facility following our institutional guidelines, and the experimental procedures were approved by the institutional review board of Chungnam National University.

### Isolation of lung endothelial cells

To purify lung endothelial cells, five 8-week-aged wild type (WT) and CRIF1 EKO (CRIF1 endothelial knockout) mice were used for our procedures. Each mouse was anesthetized with urethane (1.3 g/kg, ip), followed by exposure of the thoracic cavity. Lungs were subsequently removed from the chest cavity of mice, and then incubated with 15 ml of collagenase buffer (Dulbecco’s Modified Eagle’s Medium [DMEM; Gibco] supplemented with 1 mg/ml collagenase type I (Worthington)] in a 50-ml tube at 37°C for 40 min with gentle shaking and then filtered through a 100 μm filter. Digested cells were centrifuged for 5 min at 700 g and incubated with red blood cell lysis buffer (cat no. 00–4333; e-Biosciences, San Diego, CA, USA). The remaining cells were washed twice with phosphate-buffered saline (PBS). Subsequently, magnetic activated cell sorting (MACS) was used to isolate endothelial cells from the lung single-cell suspension. Reagents used for MACS were purchased from Miltenyi Biotec Inc. (Bergisch Gladbach, Germany) including CD31 MicroBeads (cat no. 130-097-418) and CD45 MicroBeads (cat no. 130-052-301). Endothelial cells were isolated using anti-CD45 and anti-CD31 antibody-conjugated magnetic beads, and the MACS system was run according to the manufacturer's instructions. Because CD31 is expressed on some immune cells, and endothelial cells do not express CD45, we first isolated CD45-negative cells, and then isolated CD31-positive cells from the CD45-negative cells. Finally, we obtained endothelial cells of highest purity, which were used for analysis.

### Enzyme-linked immunosorbent assay (ELISA)

Six 8-week-aged WT and CRIF1 EKO mice were anesthetized (urethane, 1.3 g/kg, ip), and blood was collected by cardiac puncture. Mouse blood samples were centrifuged for 20 min at 3000 g at room temperature, and the plasma supernatant was transferred to new tubes and frozen immediately at ‒80°C. ELISA kits for TNF-α (R&D Systems, MTA00B), IL-1β (R&D Systems, MLB00C), and IL-6 (R&D Systems, M6000B) were used, and inflammatory cytokine levels in mouse serum were detected according to the manufacturer’s protocols.

### Western blotting

To examine the signaling pathway involved, HUVECs were pretreated with 5 mM NAC or 5 uM BAY117082 (all purchased from Sigma) for 1 h and then transfected with CRIF1 (100 pmol) for 48 h. In another experiment, HUVECs were treated with βGal (30 MOI) or SIRT1 adenovirus (10, 30 MOI) for 24 h and then transfected with CRIF1 (100 pmol) for 36 h. Cultured HUVECs were harvested in 100 μL lysis buffer (20 mM Tris-HCl, pH 7.5, 150 mM NaCl, 1 mM ethylene glycol tetraacetic acid, 1% NP-40, 1% sodium deoxycholate, 2.5 mM sodium pyrophosphate, 1 mM sodium orthovanadate, 1 mM β-glycerophosphate, and a protease inhibitor cocktail). Cell lysates were prepared by centrifugation at 12,000 rpm for 15 min, and the supernatant was collected. Proteins separated by sodium dodecyl sulfate‒polyacrylamide gel electrophoresis were transferred to polyvinylidene difluoride membranes (Immobilon-PSQ, Millipore). After blocking in blocking solution (6% skim milk in TBST) for 1 h at room temperature, the membranes were incubated overnight at 4 ^o^C with the following specific primary antibodies: anti-CRIF1 (Santa Cruz Biotechnology, Santa Cruz, CA, USA), anti-SIRT1 (Santa Cruz Biotechnology, Santa Cruz, CA, USA), anti-β-actin (Sigma-Aldrich, St. Louis, MO, USA), anti-p65 (Santa Cruz Biotechnology, Santa Cruz, CA, USA), anti-PARP (Santa Cruz Biotechnology, Santa Cruz, CA, USA), anti-VCAM-1 (Santa Cruz Biotechnology, Santa Cruz, CA, USA), and anti-IκBα (Santa Cruz Biotechnology, Santa Cruz, CA, USA). The purity of the nuclear fraction was confirmed using an antibody against the nuclear marker PARP. Chemiluminescent signals were developed using Super Signal West Pico or Femto Substrate from Thermo Fisher Scientific (Waltham, MA, USA). Western blotting of 30 μg whole-cell lysates or tissue homogenate was performed similarly using appropriate primary and secondary antibodies. The membranes were treated with an appropriate peroxidase-conjugated secondary antibody, and the chemiluminescent signal was developed using Super Signal West Pico or Femto Substrate (Pierce Biotechnology, Rockford, IL, USA). Values were normalized to β-actin or PARP as loading controls.

### Histological and morphometric analyses

8-week-old WT and CRIF1 knockout mice (n = 5 for each group) were anesthetized (urethane, 1.3 g/kg, ip), and aortas were subsequently removed from the chest cavity of mice, cut into 2-mm width rings, and cultured in endothelial growth medium 2. Each aorta was treated ex vivo with β-Gal adenovirus (3.0×10^8^ pfu/well) or SIRT1 adenovirus (3.0×10^8^ pfu/well) and incubated at 37°C for 4 h, then removed the solution and incubated for another 20 h before sectioning into rings. After 24 h, aortas were fixed in 10% neutralized formalin for 16 h, washed, and embedded in paraffin. Paraffin sections were deparaffinized and rehydrated. For immunohistochemistry, deparaffinized mouse aortic ring sections were permeabilized and processed using the Vectastain Universal Quick Kit (PK-8800; Vector Laboratories, Burlingame, CA, USA). Primary antibodies against CD31 (Millipore, Temecula, CA, USA) or VCAM-1 (Santa Cruz Biotechnology, Santa Cruz, CA, USA) were used at a 1:50 dilution followed by incubation with biotinylated secondary antibody, streptavidin peroxidase solution, and nucleus staining was performed with DAPI. Axiophot microscope (Carl Zeiss, Germany) was used for the analysis of double-stained sections. To quantify VCAM-1 content, quantitative morphometric analysis was performed using Pannoramic Viewer 1.14 Software (3DHISTECH Ltd., Budapest, Hungary).

### Immunocytochemistry

HUVECs were cultured on glass coverslips in 12-well plates and transfected with (100 pmol) siRNA CRIF1 for 48 h. After washing with phosphate-buffered saline, cells were fixed with 4% (w/v) paraformaldehyde and permeabilized with 0.1% (v/v) Triton X-100. After blocking for 1 hour with PBS containing 5% (w/v) bovine serum albumin and 5% (v/v) horse serum, cells were incubated with NF-κB p65 antibody (1:500) or VCAM-1 antibody (1:500) overnight at 4°C and then labeled with Alexa Fluor 488-conjugated secondary antibody (1:500) or Fluor 647-conjugated secondary antibody (1:500) for 1 hour in the dark at room temperature. Images were obtained by fluorescence microscopy.

### Preparation of nuclear and cytosolic fractions

After transfection or treatment, nuclear and cytosolic fractions were isolated from HUVECs using the NE-PER extraction kit (Pierce, Rockford, IL, Catalogue number: 78833) according to the manufacturer’s instructions. The cells were lysed and centrifuged at 12,000 rpm for 10 min. The supernatant (cytosolic fraction) and nuclear pellet were collected. The cytosolic fraction was lysed in 20 mM/L Tris buffer (pH 7.5) containing 0.5 mM/L EDTA-Na2, 0.5 mM/L EGTA-Na2, and protease inhibitors. The supernatant fractions were collected and used for Western blotting of p65-NF-κB. The nuclear pellet was re-suspended in nuclear buffer (20 mM HEPES, pH 7.9, 0.4 M NaCl, 1 mM EDTA, 10% glycerol, 1 mM DTT, and protease inhibitors), vortexed, and centrifuged at 12,000 rpm for 20 min. The supernatant fractions were collected and used for Western blotting of p65-NF-κB and IκBα.

### Dual luciferase assay

HUVECs were plated at 2 × 10^5^/well in 6-well plates, allowed to attach overnight, and transfected with 1 μg total plasmid containing 0.99 μg/well NF-κB-luciferase reporter vector4 and 10 ng/well pCMV-pRL internal control vector (Promega) using the transfection reagent Effectene (Qiagen, Valencia, CA, USA). After transfection for 24 h, the cells were transfected with siRNA targeting CRIF1 and negative control siRNA for another 36 h, washed with ice-cold PBS, and harvested in reporter lysis buffer. After centrifugation, 20 μl of the supernatant fraction were used for measurement of dual luciferase activity using a luminometer. Luciferase activity was normalized to the protein concentration and expressed as relative luciferase activity (ratio of firefly luciferase activity to Renilla luciferase activity units).

### qPCR

Total RNA from HUVECs or lung endothelial cells were isolated using TRIzol Reagent according to the manufacturer’s protocol (Invitrogen). The RNA concentration was quantified using using a SmartSpec 3000 spectrophotometer (Bio-Rad, Hercules, CA, USA). cDNA was prepared from total RNA using the Maxime RT Premix kit (iNTRON Biotechnology, Seoul, South Korea). qPCR was performed using the Prism7000 Sequence Detection System (Applied Biosystems, Foster City, CA, USA) with the Super Script III Platinum SYBR GreenOne-Step qRT-PCR Kit (Invitrogen). The primers used for mouse VCAM-1 were sense-5’-TGAACCCAAACAGAGGCAGAG-3’ and antisense-5’-GGTATCCCAT CACTTG AG CAG-3’. The primers used for human TNFα, IL-1β, and IL-6 were as follows: TNFα sense-5’- 5′CCCAGGGACCTCTCTCTAATCA-3’ and antisense-5’-AGCTGCCCCTCAG CTTGAG-3’; IL-6 sense-5’-CCACTCACCTCTTCAGAACG-3’ and antisense-5’- CATCT TTGGAAGGTTCAGGTT G-3’; IL-1β sense-5’- 5′CCCAGGGACCTCTCTCTAATCA-3’ and antisense-5’-AGCTGCCC CTCAGCTTGAG-3’. The primers for mouse glyceraldehyde 3-phosphate dehydrogenase, used as the internal control, were as follows: sense-5’-ATGACATCAAGAAGGTGGTG-3’ and antisense-5’-CATACCAGGAAAATGAGCTTG-3’. Dissociation curves were monitored to check the aberrant formation of primer-dimers. Amplification reactions took place on the Realplex (Eppendorf). All fold changes were calculated using the 2^-ΔΔCt^ method and are expressed as means ± standard error of the mean (SEM). All experiments included three to six samples per group and time point.

### Statistical analysis

Statistical analysis was performed using SPSS (version 17.0) statistical software (SPSS Inc., Chicago, IL, USA). Differences between two groups were evaluated using t-tests. One-way analysis of variance was performed for multiple comparisons, and Tukey’s test was carried out for post-hoc analyses. Data are presented as means ± SEM. A value of P ≤ 0.05 was considered to indicate statistical significance. All data are representative of at least three independent experiments

## Results

### Endothelial CRIF1 deletion enhanced proinflammatory cytokine production and VCAM-1 expression *in vivo and in vitro*

**Fig 1:**
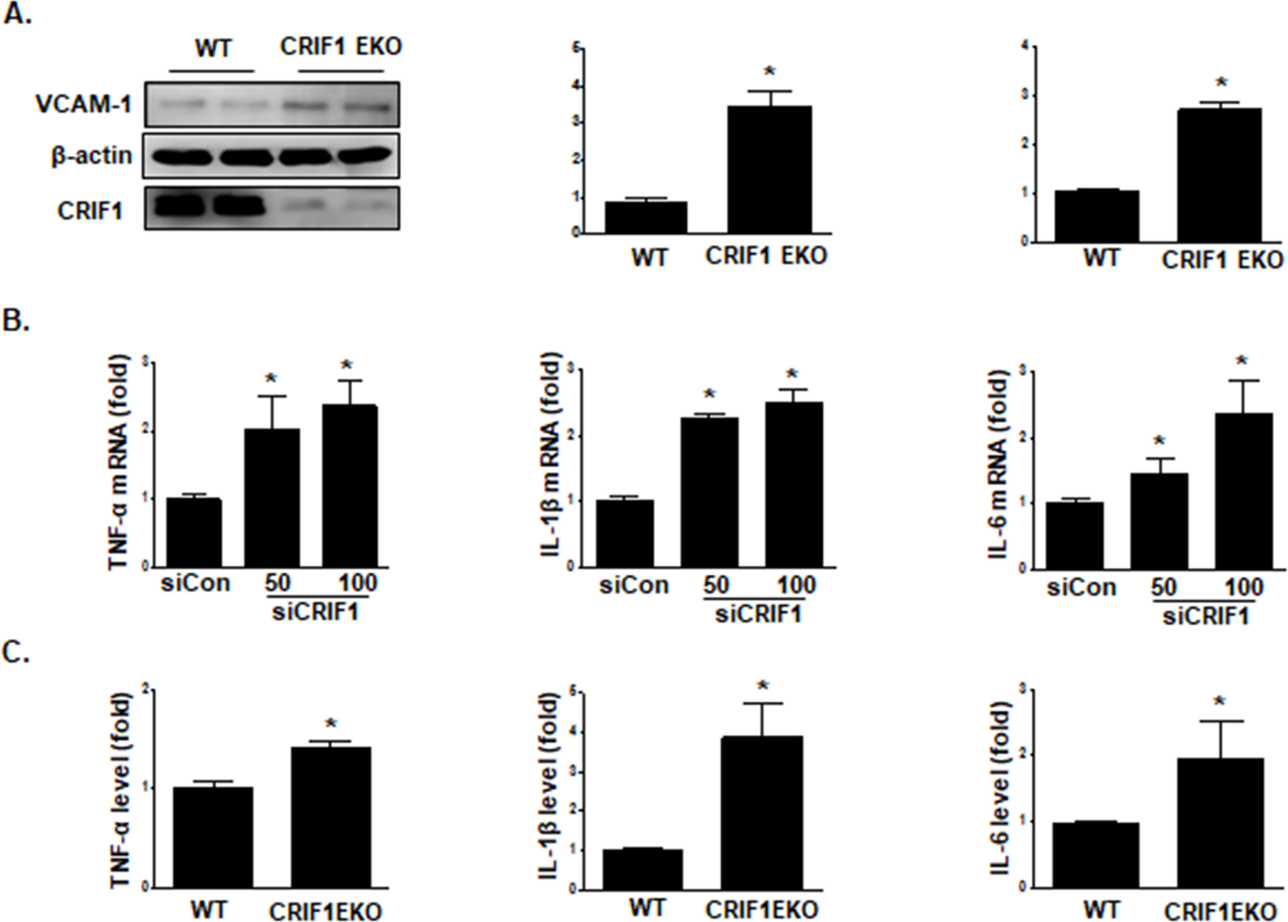
Endothelial CRIF1 deletion elevated inflammatory cytokine and VCAM-1 expression *in vivo* and *in vitro*. (A) Lung endothelial cells were isolated from WT and CRIF1 EKO mice using CD31 and CD45 beads. Endothelial VCAM-1 mRNA and protein expression was determined by western blotting and qPCR, respectively. β-actin was used as the internal control. Densitometric analysis of VCAM-1 protein levels is shown in the middle panel, and the relative mRNA expression of VCAM-1 is shown in the right panel. (B) HUVECs were transfected with CRIF1 siRNA (50 or 100 pmol) for 48 h. TNFα, IL-1β, and IL-6 mRNA levels were quantified by qPCR. (C) ELISAs of TNF-α, IL-1β, and IL-6 expression in the plasma of WT and CRIF1 EKO mice. Data are expressed as the fold change in expression compared with the control group. Western blots are representative of three independent experiments. The data are presented as means ± SEM of three independent experiments. *p < 0.05 vs. control cells.

Previously, we showed that CRIF1 deficiency upregulated ROS production and VCAM-1 expression in HUVECs [12]. To identify the role of endothelial CRIF1 in vascular inflammation, we crossed conditional CRIF1 *flox/flox* mice with *Tek-Cre* transgenic mice to generate CRIF1 endothelium-specific knockout mice (CRIF1 EKO) and then purified CD31+/CD45- endothelial cells from the lungs of WT and CRIF1 EKO mice using the MACS system. The mRNA and protein levels of VCAM-1 in CD31+/CD45- endothelial cells from CRIF1 EKO mice were significantly elevated compared with those from WT mice (Fig 1A). To reveal the mechanisms underlying the role of endothelial CRIF1 in inflammation, we first examined whether CRIF1 deletion elevates the expression of proinflammatory cytokines, including TNFα, IL-1β, and IL-6, in HUVECs. To silence CRIF1 protein expression in HUVECs, cells were transfected with 50 and 100 pmol CRIF1; we then measured the mRNA expression of inflammatory cytokine mRNA by quantitative PCR analysis. As shown in Fig 1B, the mRNA expression of TNFα, IL-1β, and IL-6 was significantly elevated in the CRIF1 deficiency group compared with the control siRNA group. In addition, plasma concentrations of these cytokines were determined using ELISA in both the WT and CRIF1 EKO mouse groups. Cytokine levels were significantly upregulated in CRIF1 EKO mice compared with WT mice, while IL-1β levels were changed extensively (Fig 1C). These findings suggest that endothelial CRIF1 deletion may induce vascular inflammation via enhanced production of proinflammatory cytokines.

### NF-κB inhibitor decreased CRIF1 deletion-induced VCAM-1 expression in HUVECs

**Fig 2:**
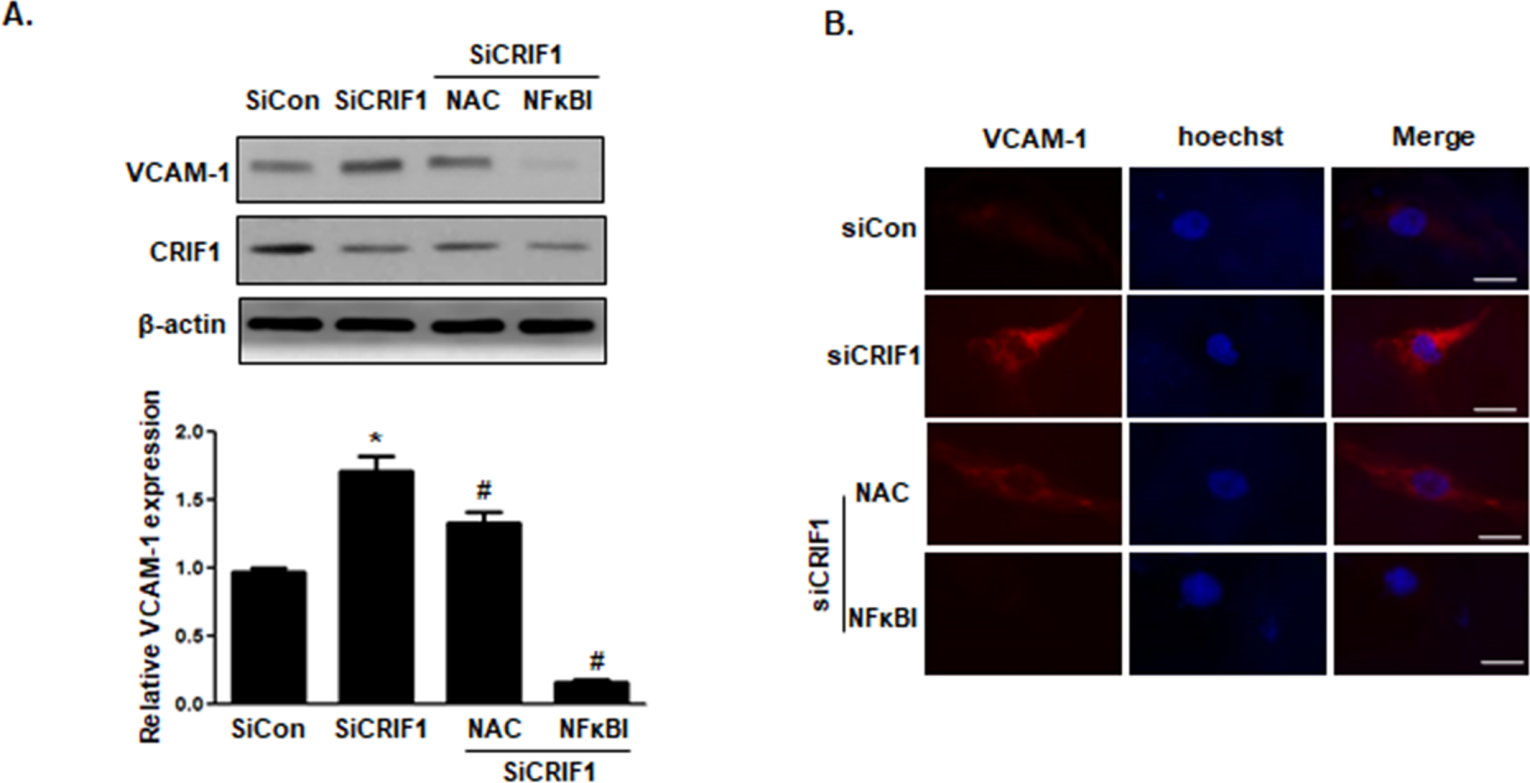
NAC and NF-κB inhibitor decreased CRIF1 deletion-induced expression of VCAM-1 in HUVECs. Endothelial HUVECs were pretreated with NAC and NF-κB inhibitor (BAY11-7082) for 1 h and then transfected with CRIF1 siRNA (50, 100 pmol) for 48 h. (A) VCAM-1 protein expression was detected by western blotting and quantified by densitometric analysis. (B) The location and protein expression of VCAM-1 (red) and Hoechst (blue) in HUVECs were examined by immunofluorescence. Scale bars indicate 20 um. All western blots are representative of three independent experiments. The data are presented as means ± SEM of three independent experiments. *p < 0.05 vs. control cells. ^#^p < 0.05 vs. CRIF1 siRNA cells.

We previously demonstrated that VCAM-1 mRNA and protein expression in both CRIF1 siRNA-transfected HUVECs and CRIF1 EKO lung endothelial cells showed significantly higher expression compared with control siRNA and WT mice, respectively. There is abundant evidence indicating that the NF-κB signaling pathway plays a crucial role in the regulation of inflammatory cytokines, including TNF-α, IL-1β, and IL-6, and adhesion molecules, including ICAM-1 and VCAM-1 [20, 21]. Thus, to further elucidate the potential mechanisms underlying CRIF1 in endothelial inflammation, we pretreated cells with the antioxidant N-acetyl-cysteine (NAC), and a NF-κB inhibitor (BAY11-7082) for 1 h prior to transfecting cells with CRIF1. As shown in Fig 2A, NAC and NF-κB inhibitor extensively decreased CRIF1 downregulation-induced VCAM-1 expression. Immunocytochemistry showed the same pattern as the western blot data (Fig 2B), suggesting that ROS and NF-κB are upstream signaling molecules involved in the regulation of VCAM-1 expression. Therefore, we hypothesized that CRIF1 deficiency may increase transcription factor NF-κB activation in HUVECs.

### CRIF1 deletion stimulated activation of the NF-κB signaling pathway in HUVECs

**Fig 3:**
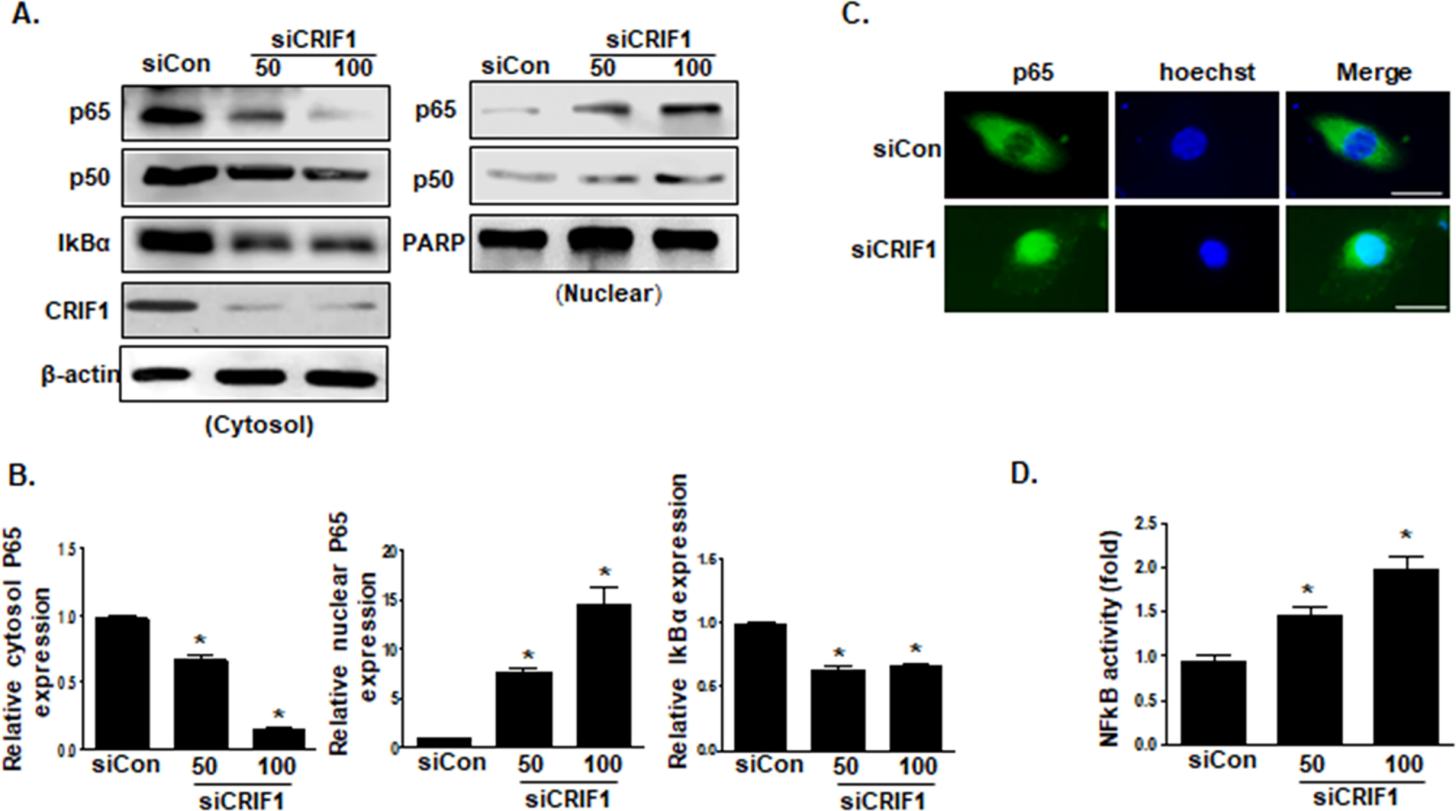
CRIF1 deletion stimulated the activation of the NF-κB signaling pathway in HUVECs. (A) HUVECs were transfected with CRIF1 (50 or 100 pmol) for 48 h, cytosolic and nuclear fractions were isolated, and p65 and IκBα protein levels in the cytoplasm and nucleus were detected by western blotting. (B) p65 and IκBα protein levels were quantified by densitometric analysis. (C) Representative immunofluorescence images of p65 (green) and Hoechst (blue) staining. Scale bars indicate 20 um. (D) NF-κB activity was measured using a dual luciferase reporter gene assay in HUVECs transfected with CRIF1 siRNA (50 and 100 pmol) for 48 h. The graph presents the fold changes in NF-κB activation compared with that in the control. All western blots are representative of three independent experiments. The data are presented as means ± SEM of three independent experiments. *p < 0.05 vs. control cells.

NF-κB, an important transcription factor, regulates inflammatory responses by promoting leukocyte adhesion to the endothelium and increasing adhesion molecules expression [22]. In quiescent cells, the NF-κB p65 transcription factor is tightly associated with its inhibitor IκBα in the cytoplasm. Under stimulation, IκBα is degraded together with p65 accumulation and then translocated to the nucleus, stimulating inflammatory gene transcription. We demonstrated that an NF-κB inhibitor completely blunted CRIF1 deficiency-induced VCAM-1 protein expression (Fig 2). Thus, we further examined the nuclear translocation of p65 using western blotting and immunofluorescence. As shown in Fig 3A, CRIF1 siRNA-treated cells (50 and 100 pmol) showed evidence of IκBα degradation and p65 translocation to the nucleus. Consistent with these results, immunofluorescence staining revealed that p65 was localized predominantly in the cytoplasm in controls, while CRIF1 knockdown strongly induced p65 translocation to the nucleus (Fig 3B). Furthermore, elevation of NF-κB activity was confirmed in CRIF1-silenced cells in a dual luciferase reporter assay. Taken together, CRIF1 deletion activated the NF-κB signaling pathway in HUVECs.

### SIRT1 mediated CRIF1 deficiency-induced NF-kB activation in HUVECs

**Fig 4:**
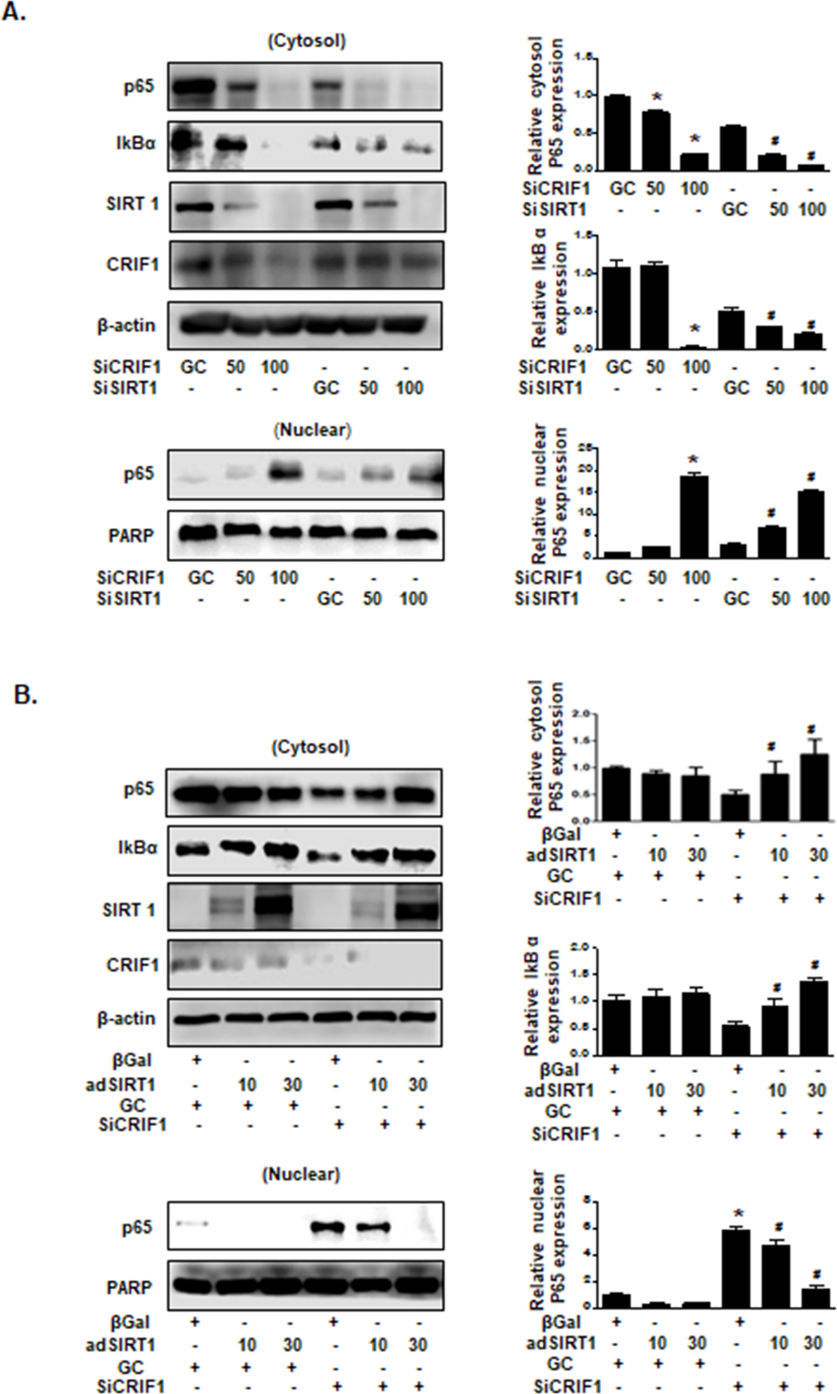
SIRT1 mediated CRIF1 deficiency-induced NF-κB activation in HUVECs. (A) HUVECs were transfected with CRIF1 siRNA (50 and 100 pmol) and SIRT1 siRNA (50 and 100 pmol) for 48 h, and the cytosolic and nuclear fractions were isolated. The protein expression levels of cytosolic p65 and IκBα and nuclear p65 were detected by western blotting. p65 and IκBα protein levels were quantified by densitometric analysis (right panel). (B) HUVECs were infected with β-Gal and SIRT1 adenovirus for 24 h, followed by transfection with CRIF1 siRNA (100 pmol) for 36 h. Cytosolic p65 and IκBα and nuclear p65 protein expression levels were quantified by densitometric analysis (right panel). All western blots are representative of three independent experiments. The data are presented as means ± SEM of three independent experiments. ^#^p < 0.05 vs. CRIF1 control cells. *p < 0.05 vs. SIRT1 control cells.

We have shown that CRIF1 deletion-induced NF-κB activation is mediated by p65 translocation from the cytoplasm to the nucleus, and SIRT1 is an important regulator of cellular energy metabolism. Considerable research has indicated antagonistic crosstalk between SIRT1 and the NF-κB signaling pathway [15]. SIRT1 was shown to inhibit p65 translocation to the nucleus and consequently to suppress NF-κB-dependent gene expression [23]. Conversely, SIRT1 deficiency results in NF-κB activation and a subsequent inflammatory response [17, 18, 24]. Moreover, our previous studies have demonstrated that CRIF1 deficiency downregulated SIRT1 levels and upregulated eNOS acetylation [19]. To examine whether decreased SIRT1 protein expression induced NF-κB activation in HUVECs, we transfected HUVECs with 50 and 100 pmol SIRT1 siRNA and CRIF1 siRNA with an incubation period of 48 h, respectively. Western blot analysis of the cytosolic and nuclear fractions showed that both downregulation of SIRT1 and CRIF1 decreased p65 and IκBα expression in the cytoplasm and increased p65 protein expression in the nucleus compared with the siRNA control (Fig 4A), illustrating that CRIF1-induced SIRT1 inhibition may be an essential factor triggering NF-κB activation. Subsequently, to investigate whether SIRT1 overexpression suppresses NF-κB activation, we pretreated cells with SIRT1 adenovirus prior to CRIF1 siRNA transfection and subsequently detected p65 protein expression in both cytosolic and nuclear fractions. As shown in Fig 4B, SIRT1 overexpression also induced a dose-dependent reduction in CRIF1 deficiency-stimulated NF-κB activation. These findings strongly suggest that SIRT1-mediated cytoplasmic decreases in p65 and IκBα proteins in CRIF1-deficient cells strongly affected NF-kB activation in endothelial cells.

### SIRT1 mediated CRIF1 deletion-induced expression of inflammatory cytokines and VCAM-1

**Fig 5:**
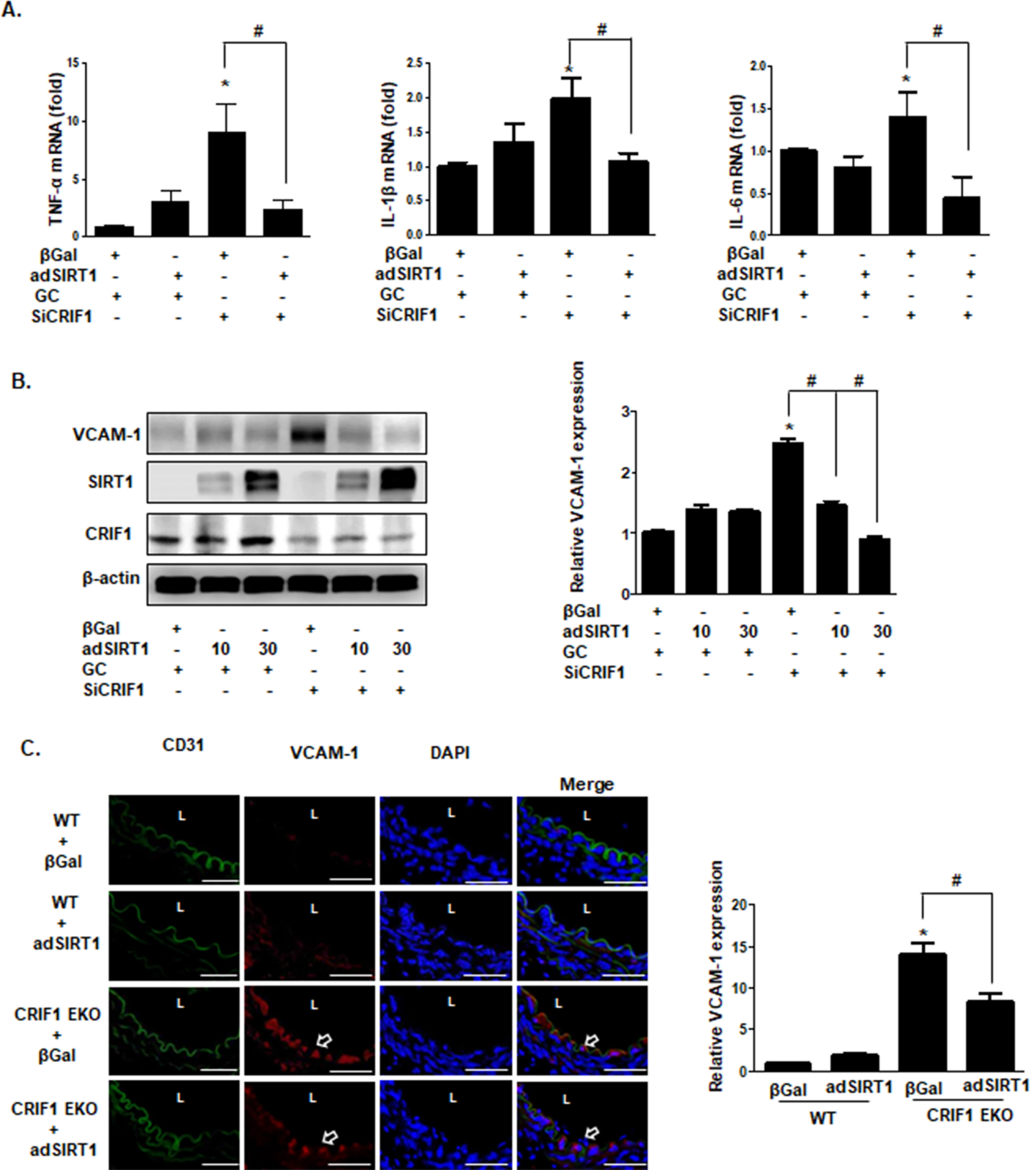
SIRT1-mediated CRIF1 deletion-induced inflammatory cytokine and VCAM-1 expression. (A) HUVECs were infected with β-Gal and SIRT1 adenovirus for 24 h, followed by transfection with CRIF1 (100 pmol) siRNA for 36 h. TNFα, IL-1β, and IL-6 mRNA levels were quantified using qPCR. (B) Whole cell lysates were collected and analyzed for VCAM-1 protein expression by western blotting. VCAM-1 protein levels were quantified by densitometric analysis (right panel). (C) The aorta from WT and CRIF1 EKO mice were infected with β-Gal (control) or SIRT1 adenovirus and incubated for 24 h. Representative immunohistochemical staining images of CD31 (green) and VCAM-1 (red). Scale bars indicate 50 um. Arrowhead indicates VCAM-1 expression. L indicates lumen. Quantified VCAM-1 expression was normalized to the β-Gal (WT) control group (right panel). n = 5 per group. All western blots are representative of three independent experiments (A, B). The data are presented as means ± SEM of three independent experiments. *p < 0.05 vs. control cells or WT mice. ^#^p < 0.05 vs. CRIF1 cells or CRIF1 EKO mice.

As NF-kB plays an important role in regulating inflammatory cytokine production and VCAM-1 induction, we examined whether adenovirus-mediated SIRT1 overexpression blocks CRIF1-induced TNFα, IL-1β, IL-6 and VCAM-1 expression in endothelial cells. We pretreated cells with SIRT1 adenovirus following CRIF1 knockdown to investigate TNFα, IL-1β, and IL-6 mRNA expression. As shown in Fig 5A, overexpression of SIRT1 significantly inhibited CRIF1 deficiency-induced upregulation of TNFα, IL-1β, and IL-6 mRNA levels in HUVECs. Furthermore, increased VCAM-1 protein expression was significantly reduced by overexpression of SIRT1 in CRIF1-silenced cells, suggesting that SIRT1 mediates CRIF1 deficiency-induced VCAM-1 expression (Fig 5B). To further explore the functional role of SIRT1 in the CRIF1-dependent inflammation *in vivo*, mice aortic rings were infected with SIRT1 adenovirus (3x10^8^) for 24 h and then immunostained for both VCAM-1 and the endothelial marker CD31. As shown in Fig 5C, colocalization of VCAM-1 and CD31 expression was significantly reduced in CRIF1 EKO/SIRT1-adenovirus mice compared with CRIF1 EKO mice, suggesting that SIRT1 is an important modulator of CRIF1 EKO-induced endothelial inflammation.

## Discussion

**Fig 6:**
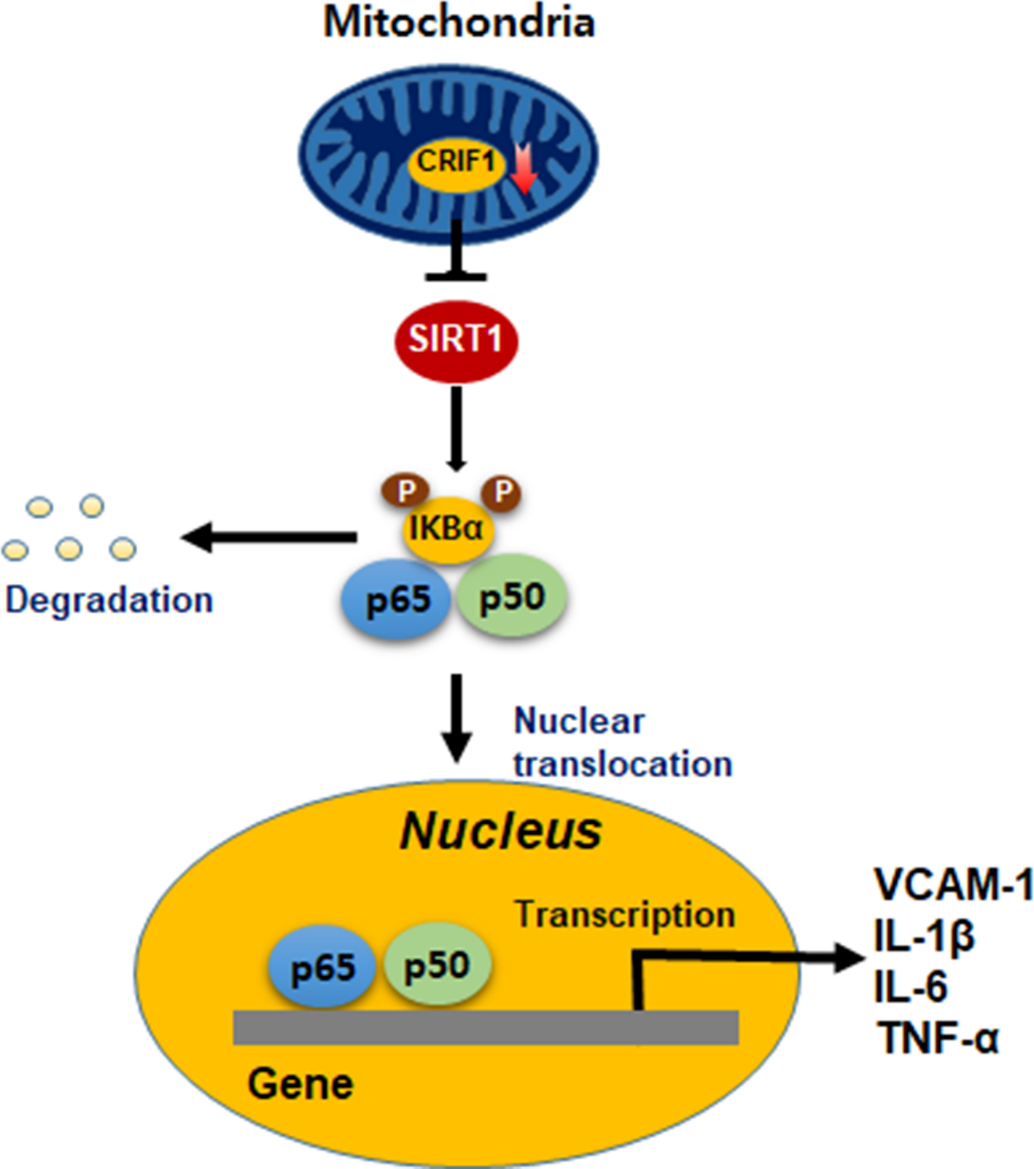
Schematic model summarizing the underlying mechanisms and function of CRIF1 on inflammation.

Vascular function and structural alterations may contribute to hypertension, atherosclerosis, diabetes mellitus, and other cardiovascular diseases [25, 26]. The endothelium regulates vascular tone and permeability by influencing the vascular function and remodeling [1]. Therefore, endothelial inflammation occurs in association with endothelial dysfunction and vascular remodeling, which leads to several cardiovascular diseases such as hypertension and atherosclerosis [9]. There is considerable evidence indicating that mitochondrial dysfunction contributes to endothelial dysfunction, which leads to oxidative stress and inflammation, resulting in vascular dysfunction and other diseases [5, 6]. Moreover, accumulating evidence supports that mitochondrial dysfunction generates inflammatory responses [27, 28]. CRIF1 is a mitochondrial protein and interacts with the 39S subunit of the mitoribosome, which is essential for the synthesis and insertion of OXPHOS polypeptides [10]. We have demonstrated that CRIF1 deficiency reduced OXPHOS expression accompanied by increased mitochondrial ROS expression, which decreased SIRT1 expression, eNOS activity and vasomotor function [19]. However, we have not investigated whether CRIF1 downregulation in endothelial cells is capable of triggering inflammatory responses via mitochondrial-derived ROS or other mechanisms. The results of this study demonstrated that CRIF1 deficiency- elevated the levels of inflammatory mediators, including VCAM-1, TNF-α, IL-1β and IL-6 via the activation of NF-κB pathway (Fig 6). Furthermore, SIRT1 overexpression led to recovery of these inflammatory mediators by suppressing NF-κB activity.

Inflammation of the vascular wall is an important activator to atherosclerosis, hypertension and other cardiovascular diseases. Leukocyte adhesion molecules, such as VCAM-1, ICAM-1, and E-selectin are the main endothelial adhesion molecules, which are crucial for the monocyte accumulation in the arterial intima [29, 30] and the firm adhesion of leukocytes to the endothelium, therefore participating in the inflammatory response in the vascular wall. Monocytes transform into macrophages and secret proinflammatory cytokines such as TNF-α, IL-1β, and IL-6 to participate in the initiation and progression of inflammation. VCAM-1 plays a dominant role in the migration of monocytes to the vessel wall [31] and in the initiation of inflammation and atherosclerosis [32]. Our data showed that endothelium-specific deletion of CRIF1 resulted in enhanced mRNA and protein expression of VCAM-1 in CRIF1 EKO mice (Fig 1A), in accordance with the results in HUVECs. In addition, the production of proinflammatory cytokines (TNF-α, IL-1β, and IL-6) was markedly elevated in endothelial-specific CRIF1 knockout mice and CRIF1-silenced endothelial cells, respectively (Fig 1). Taken together, CRIF1 deficiency induced expression of inflammatory mediators including VCAM-1, TNF-α, IL-1β, and IL-6. It is unknown whether CRIF1 knockdown- induced endothelial inflammation is involved in atherosclerosis and other cardiovascular diseases. Further investigation is necessary to determine the mechanism and signaling pathway in CRIF1 deletion-induced endothelial inflammation.

The NF-κB pathway is the major signaling pathway involved in initiating and sustaining vascular inflammation [33], which can be triggered by several stimuli, including oxidative stress, inflammatory cytokines, radiation, and other stimulatory factors [34, 35]. Previously, we demonstrated that CRIF1 deficiency produced mitochondrial ROS, triggering inflammatory mediators production. The proinflammatory cytokines such as TNF-α and IL-1β are not only targeted by NF-κB but also stimulate NF-κB via their receptors [36]. In addition, our previous studies have indicated that downregulation of CRIF1 severely disturbed mitochondrial OXPHOS complexes and enhanced mitochondrial ROS production [12], which may also lead to activation of the redox-sensitive NF-κB transcription factor. Hence, we hypothesize that both cytokines and ROS production in endothelial cells with CRIF1 downregulation may lead to NF-κB activation and continue to augment cellular inflammation. In order to investigate whether ROS and the NF-κB signaling pathway are associated with CRIF1 deficiency-induced inflammation, we pretreated endothelial cells with the antioxidant NAC and NF-κB inhibitor, and then detected the expression of VCAM-1 in endothelial cells with CRIF1 deletion. Our western blot and immunochemistry data showed that the antioxidant NAC and NF-κB inhibitor significantly decreased CRIF1 deficiency-induced VCAM-1 expression, which indicates that CRIF1 deletion exerted inflammatory effects in endothelial cells mediated mainly by the NF-κB pathway (Fig 2). Consistent with these results, the level of activated NF-κB was significantly upregulated in CRIF1 siRNA cells compared with control cells. Taken together, these data strongly support that CRIF1 stimulate NF-κB activation in endothelial cells. However, NAC pretreatment in CRIF1 siRNA cells did not cause a severe decrease in VCAM-1 expression, compared with NF-κB inhibitor. NAC, a ROS scavenger, mainly exerts its antioxidant action by promoting glutathione (GSH) synthesis, which is produced in the cytosol and then transported into the mitochondria. Previous research has shown that despite the increase in cytosolic GSH levels by NAC treatment, NAC did not increase mitochondrial GSH levels, suggesting that the NAC was unable to scavenge mitochondrial oxidative stress [37]. Therefore, our observations suggest that CRIF1 deficiency-induced mitochondrial ROS production, and not cytosolic ROS production, maybe the main trigger of inflammation by increasing NF-κB activation.

SIRT1 is a member of the sirtuin family, which encodes nicotinamide adenine dinucleotide (NAD)^+^-dependent deacetylases, and mainly modifies histones and transcription factors via deacetylation [38]. Recent studies have reported that SIRT1 inhibits the NF-κB signaling pathway via deacetylation of the RelA/p65 subunit of the NF-κB complex [23, 39]. However, plentiful evidence indicates that NF-κB signaling also decreases SIRT1 activity, demonstrating antagonistic crosstalk between SIRT1 and NFkB. Zeng *et al*. reported that SIRT1 knockdown induced hepatic inflammation by increasing the activation of NF-κB [40]. Breitenstein *et al*. reported that SIRT1 inhibition enhanced NF-κB activation via acetylation of Lys310 NF-κB /p65 in human aortic endothelial cells [41]. To investigate the effect of SIRT1 deletion on NF-κB in HUVECs, we only silenced SIRT1 expression via transfection of SIRT1 siRNA and then evaluated p65 expression in both cytosol and nuclear fractions. Our results demonstrated that SIRT1 inhibition significantly stimulated NF-κB activation, which showed the same results with CRIF1 deletion-induced NF-κB activation (Fig 4A). Furthermore, SIRT1 has been identified to bind to eNOS and it may play a critical role in promoting endothelium-dependent vascular relaxation by deacetylating eNOS, stimulating eNOS activity and increasing endothelial NO production [42]. Our previous results also demonstrated that CRIF1 deficiency-induced mitochondrial ROS downregulated SIRT1 levels by increasing eNOS acetylation and decreasing NO production [19]. However, SIRT1 overexpression led to recovery of impaired vascular dysfunction by reducing eNOS acetylation and elevating its bioavailability, which suggests that SIRT1 is a key modulator of CRIF1 deficiency-induced endothelial dysfunction. Next, we investigated whether SIRT1 overexpression possesses anti-inflammatory effects by inhibiting NF-κB activation. We pretreated cells with SIRT1 adenovirus followed by CRIF1 knockdown to investigate the expression of inflammatory mediators including VCAM-1, TNF-α, IL-1β, IL-6, and NF-κB. As shown in Fig 5, the expression of inflammatory mediators was obviously inhibited by overexpression of SIRT1 in CRIF1 knockdown cells. Hence, our data strongly indicate that SIRT1 plays a vital role in CRIF1 deficiency-related endothelial inflammation, which maybe a novel therapeutic target for the treatment of vascular diseases.

In summary, our study demonstrates that CRIF1 deficiency promoted endothelial cell inflammation by increasing expression of the adhesion molecule VCAM-1, elevating levels of inflammatory cytokines including TNF-α, IL-1β, and IL-6, and activating the transcription factor NF-κB. Overexpression of SIRT1 led to recovery of the CRIF1 deficiency-induced inflammatory response in endothelial cells. Thus, SIRT1 maybe a novel therapeutic target for the treatment of vascular inflammatory diseases.

## Supporting information

S1 Fig Endothelial CRIF1 deletion elevated the protein expression of VCAM-1 in mice heart tissues. (A) Western blot analysis with total tissue lysate from WT and CRIF1 EKO mice hearts. α-Tubulin was used as the internal control. (B) The expression level of VCAM-1 was quantified by densitometric analysis using image J. (right panel, values are means ± SEM, *p < 0.05). (n = 4 per group).
(TIF)
