# Supplementary figures and images for: CR6 interacting factor 1 deficiency promotes endothelial inflammation by SIRT1 downregulation
Source: PLoS One. 2018 Feb 23;13(2):e0192693. doi: 10.1371/journal.pone.0192693 (PMC5825004; doi:10.1371/journal.pone.0192693)

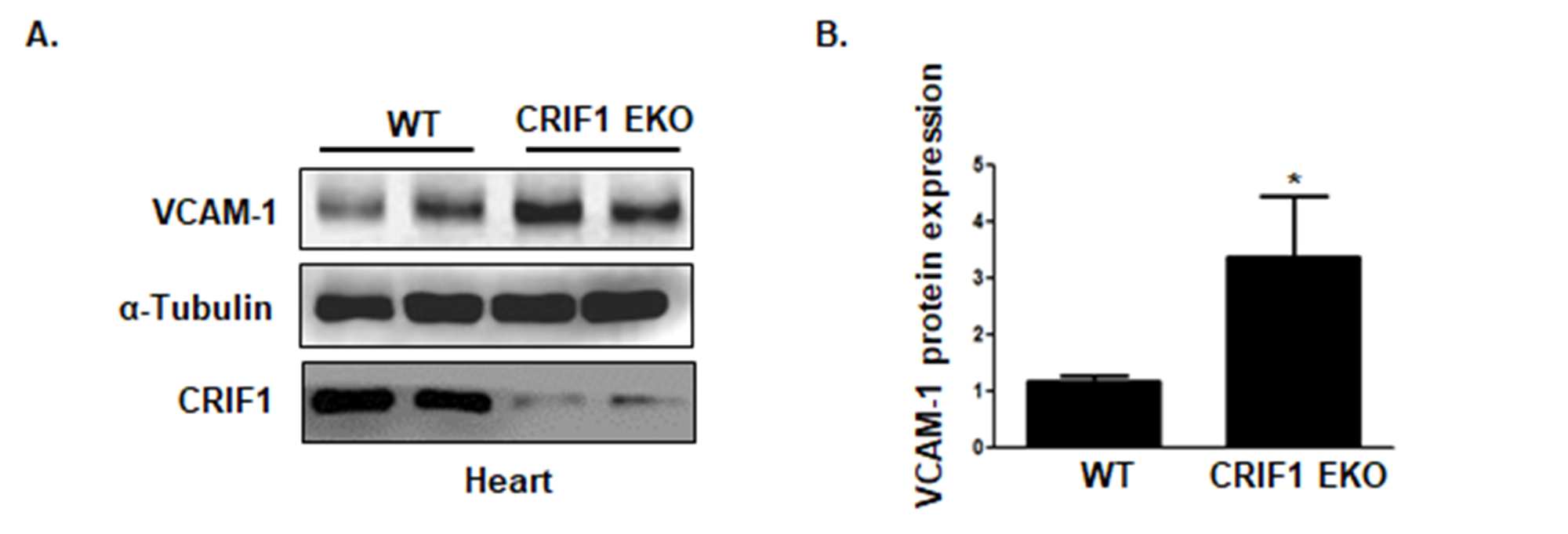

Supplement: S1 Fig — (A) Western blot analysis with total tissue lysate from WT and CRIF1 EKO mice hearts. α-Tubulin was used as the internal control. (B) The expression level of VCAM-1 was quantified by densitometric analysis using image J. (right panel, values are means ± SEM, *p < 0.05). (n = 4 per group). (TIF) [file pone.0192693.s001.tif]
